# Rapamycin weekly maintenance dosing and the potential efficacy of combination sorafenib plus rapamycin but not atorvastatin or doxycycline in tuberous sclerosis preclinical models

**DOI:** 10.1186/1471-2210-9-8

**Published:** 2009-04-15

**Authors:** Nancy Lee, Chelsey L Woodrum, Alison M Nobil, Aubrey E Rauktys, Michael P Messina, Sandra L Dabora

**Affiliations:** 1 Translational Medicine Division, Department of Medicine, Brigham & Women's Hospital, Karp Building, Boston, MA, USA

## Abstract

**Background:**

Tuberous sclerosis complex (TSC) is an autosomal dominant tumor suppressor syndrome, characterized by hamartomatous growths in the brain, skin, kidneys, lungs, and heart, which lead to significant morbidity. TSC is caused by mutations in the *TSC1 *or *TSC2 *genes, whose products, hamartin and tuberin, form a tumor suppressor complex that regulates the PI3K/Akt/mTOR pathway. Early clinical trials show that TSC-related kidney tumors (angiomyolipomas) regress when treated with the mammalian target of rapamycin (mTOR) inhibitor, rapamycin (also known as sirolimus). Although side effects are tolerable, responses are incomplete, and tumor regrowth is common when rapamycin is stopped. Strategies for future clinical trials may include the investigation of longer treatment duration and combination therapy of other effective drug classes.

**Results:**

Here, we examine the efficacy of a prolonged maintenance dose of rapamycin in *Tsc2*^+/- ^mice with TSC-related kidney tumors. Cohorts were treated with rapamycin alone or in combination with interferon-gamma (IFN-g). The schedule of rapamycin included one month of daily doses before and after five months of weekly doses. We observed a 94.5% reduction in kidney tumor burden in *Tsc2*^+/- ^mice treated (part one) daily with rapamycin (8 mg/kg) at 6 months ≤ age < 7 months, (part 2) weekly with rapamycin (16 mg/kg) at 7 months ≤ age < 12 months, and (part 3) daily with rapamycin (8 mg/kg) at 12 months ≤ age < 13 months; but we did not observe any improvement with combination IFN-g plus rapamycin in this study. We also used a *Tsc2*^-/- ^subcutaneous tumor model to evaluate other classes of drugs including sorafenib, atorvastatin, and doxycycline. These drugs were tested as single agents and in combination with rapamycin. Our results demonstrate that the combination of rapamycin and sorafenib increased survival and may decrease tumor volume as compared to rapamycin treatment alone while sorafenib as a single agent was no different than control. Atorvastatin and doxycycline, either as single agents or in combination with rapamycin, did not improve outcomes as compared with controls.

**Conclusion:**

Our results indicate that prolonged treatment with low doses of mTOR inhibitors may result in more complete and durable TSC-related tumor responses, and it would be reasonable to evaluate this strategy in a clinical trial. Targeting the Raf/Mek/Erk and/or VEGF pathways in combination with inhibiting the mTOR pathway may be another useful strategy for the treatment of TSC-related tumors.

## Background

Tuberous sclerosis complex (TSC) is a fairly common inherited tumor suppressor syndrome, characterized by the development of hamartomas in the brain, skin, kidneys, lungs, heart and other organs [1,2]. There is significant morbidity due to a variety of clinical issues that occur at high frequency including epilepsy, cognitive and/or behavioral impairments, kidney disease, pulmonary lymphangioleiomyomatosis (LAM), disfiguring facial angiofibromas, and other manifestations [2-5].

*TSC1 *and *TSC2*, which code for hamartin and tuberin respectively, have been identified as the disease genes of TSC [6,7]. The two gene products form a tumor suppressor complex that regulates a conserved cellular signaling pathway (PI3K/Akt/mTOR) that mediates protein synthesis and cell proliferation [8-11]. Tuberin's GTPase-activation of Rheb (the small GTPase Ras homologue enriched in brain) is responsible for the tumor suppressor effect of the tuberin-hamartin complex. Rheb in turn directly regulates the mammalian target of rapamycin complex 1 (mTORC1) in the PI3K/Akt/mTOR pathway [7,12,13]. When the hamartin-tuberin complex is not functional, elevated levels of active Rheb (Rheb-GTP) constitutively activate mTOR, ultimately resulting in abnormal protein translation. This in turn causes increased cell growth, proliferation, and survival [9-11,14].

Rapamycin (Rapamune™ or sirolimus, Wyeth, Madison, NJ), an FDA-approved mTOR inhibitor for immunosuppression following kidney transplantation, has been shown to ameliorate disregulated mTOR signaling in cells that lack normal hamartin or tuberin [15]. Furthermore, rapamycin and some of its analogs have successfully treated TSC-related tumors, seizures, and cognitive defects in relevant rodent disease models [16-22]. Rapamycin treatment was also effective in reducing TSC-related kidney angiomyolipomas with tolerable side effects in human clinical trials [23,24], and tumor regression was observed in a case series of TSC patients with brain tumors (subependymal giant cell tumors, also known as SGCTs or SEGAs) who were treated with off-label rapamycin [25]. There are several rapamycin analogs (CCI-779, RAD001, and AP23575) that are also under investigation as anti-tumor agents [26,27]. One of these, CCI-779 (Torisel™ or temsirolimus, Wyeth, Madison, NJ), has been FDA-approved for the treatment of advanced renal cell carcinoma [28].

While rapamycin effectively reduces the size of many TSC-associated tumors in humans, tumor regression does not occur in all cases and tumor regrowth is generally observed with the cessation of treatment [23-25]. Although the response results in early human trials are encouraging, it is possible that a longer term use of rapamycin may be more effective. Identification of other active drugs is also of interest to improve the response rate and/or durability of response. There is some evidence that other drug classes, including inhibitors of VEGF signaling, interferon gamma (IFN-g), HMG-CoA reductase inhibitors, and MMP inhibitors may be useful in treating TSC and/or LAM.

There is increasing evidence that VEGF signaling plays an important role in the pathogenesis of TSC and LAM. Brain, kidney and skin tumors associated with TSC are known to be vascular [29], and TSC2 loss is associated with elevated levels of HIF and VEGF in cultured cells [30]. Furthermore, in recent biomarker studies of the VEGF family, serum levels of VEGF-D were found to be significantly elevated in patients with sporadic or TSC-associated LAM as compared with healthy controls and patients with other pulmonary illnesses [31,32]. The importance of VEGF signaling in TSC and LAM suggests that combination therapies that aim to inhibit mTOR signaling along with disrupting VEGF signaling may be more successful than single agents. Sorafenib (also known as BAY 43-9006 and Nexavar™) is an oral multi-targeted kinase inhibitor that inhibits VEGFR-1, VEGFR-2, and VEGFR-3 in addition to the Raf/Mek/Erk pathway, PDGFR, FLT-3, and c-KIT [33,34]. It is also FDA-approved for the treatment of advanced renal cell carcinoma and advanced hepatocellular carcinoma. As a result of its inhibitory effects on angiogenic and tumorigenic molecular targets, sorafenib may be useful for treating TSC-related tumors.

The cytokine interferon-gamma (IFN-g) is another candidate therapeutic agent for the treatment of TSC because the presence of a high-expressing IFN-g allele has been linked to significantly reduced kidney tumor burdens in *Tsc2*^+/- ^mice relative to the tumor burden in the kidneys of *Tsc2*^+/- ^mice with normal IFN-g levels [35]. Furthermore, we found an association between the presence of a high-expressing IFN-g allele and reduced frequency of kidney angiomyolipomas in a cohort of human TSC patients [36]. IFN-g has also shown to be effective as a single agent in the treatment of TSC-related lesions in mouse models when IFN-g treatment is initiated while tumors are small and given for a long duration [18,19]. Recently, however, we observed that a short term course of IFN-g treatment in combination with CCI-779 did not significantly reduce kidney disease in *Tsc2*^+/- ^mice when treatment was used to treat larger tumors [20]. As such, the clinical utility of treating TSC-related tumors with the combination of IFN-g plus an mTOR inhibitor is still unclear.

Statins and MMP inhibitors are drug classes of interest because there is some evidence that they may be useful therapeutic agents for TSC. In a recent study, atorvastatin was found to inhibit the proliferation of *Tsc2*^-/- ^mouse embryo fibroblasts while also inhibiting constitutive phosphorylation of mTOR, S6 kinase, and S6 in *Tsc2*^-/- ^cells [37]. The antibiotic, doxycycline, is an MMP inhibitor that has been shown in a case report to reduce MMP levels in urine from a LAM patient. Furthermore, reduction in urine MMP levels in that case correlated with improvement of pulmonary function [38]. There is also some *in vitro *data suggesting that doxycycline inhibits MMP activity and invasiveness of cells isolated from LAM tissue [39].

We completed a series of preclinical studies in an effort to address issues relevant to making decisions regarding the next generation of clinical trials for TSC and/or LAM. Since mutations in *TSC2 *are more common and more severe compared to mutations in *TSC1 *[4], we used *TSC2 *mouse models for these studies. The *Tsc2*^+/- ^mouse is genetically similar to most humans with TSC, and they develop age-related kidney tumors that mimic important aspects of TSC-related kidney disease. We also used a *Tsc2*^-/- ^subcutaneous tumor model that reflects the loss of heterozygosity (LOH) observed in TSC-related kidney and brain tumors [40,41] as a generic model for TSC-related tumors. Specifically, we investigated the efficacy of rapamycin and rapamycin plus IFN-g using a dosing schedule that included a prolonged duration of weekly maintenance therapy using the *Tsc2*^+/- ^kidney tumor model. We also evaluated the utility of a VEGF pathway inhibitor (sorafenib), a HMG-CoA reductase inhibitor (atorvastatin), and an MMP inhibitor (doxycycline) using the subcutaneous *Tsc2*^-/- ^tumor model. These studies on new drug classes were done in the *Tsc2*^-/- ^subcutaneous tumor model because it is a relatively high throughput preclinical model relevant to TSC and/or LAM. All drugs were tested as single agents and in combination with rapamycin.

## Methods

### Treatment of *Tsc2*^+/- ^mice with IFN-g and rapamycin

The *Tsc2*^+/- ^mouse is heterozygous for a deletion of exons 1–2 as previously described [42]. The *Tsc2*^+/- ^cohort used in this experiment was obtained by crossing these *Tsc2*^+/- ^mice with wild-type C57BL/6 mice. In order to avoid bias due to strain variation, sibling littermates were used as controls. *Tsc2*^+/- ^mice were assigned to one of three cohorts: rapamycin 8 mg/kg IP, rapamycin 8 mg/kg plus IFN-g 20,000 units IP, and untreated. All mice receiving drug therapy were treated in three consecutive parts: In part one, mice were treated daily (5 days/week, Monday through Friday) for one month (6 months ≤ age < 7 months) with their assigned treatments by intraperitoneal injection (IP). In part two, all mice in both the rapamycin and rapamycin plus IFN-g cohorts stopped their assigned daily treatment and started a weekly 16 mg/kg maintenance dose of rapamycin for five months (7 months ≤ age < 12 months). In the final part, all mice restarted the same treatment they received from 6–7 months of age for one final month (see Table 1). The two month-long periods of daily rapamycin treatment before and after the maintenance treatment were included so that we can compare the results of this study with our previous preclinical studies that also include a total of two months of daily treatment without the weekly maintenance treatment phase. All mice were euthanized at 13 months of age according to institutional animal care guidelines. We evaluated kidney disease at 13 months in this experiment instead of 12 months in prior studies [18,20] because kidney disease severity is likely to be higher in older mice, and we reasoned that this may allow us to better detect small differences between treatment groups. The severity of kidney disease was determined in all animals using quantitative histopathology as described below.

**Table 1:** Summary of *Tsc2*^+/- ^results from this study and Messina et al., 2007

	Untreated	Rapamycin	Combination rapamycin plus IFN-g		Untreated	CCI-779 (6–8 months)	CCI-779 (10–12 months)
Number of mice (n)	12	24	24		12	12	12
Tumor score per kidney (ave. ± Std. Err.)	15.00 ± 2.01	0.83 ± .018	0.77 ± 0.20		9.95 ± 1.59	6.00 ± 1.01	3.54 ± 0.76
Percent decrease in tumor score per kidney with treatment	-	94.5%	94.9%		-	39.7%	64.4%
Age of kidney tumor analysis	13 months	13 months	13 months		12 months	12 months	12 months
P value (vs. untreated)	-	P < 0.0001	P < 0.0001		-	-	-
P value (vs. rapamycin)	P < 0.0001	na	P = 0.82		P < 0.0001*	P < 0.0001*	P < 0.0001*

Dose of daily rapamycin (5 days/week)	-	8 mg/kg per day	8 mg/kg per day		-	-	-
Ages, daily rapamycin treatment	-	6–7 months and 12–13 months	6–7 months and 12–13 months		-	-	-

Dose of daily IFN-g (5 days/week)	-	-	20,000 IU		-	-	-
Ages, daily IFN-g treatment	-	-	6–7 months and 12–13 months		-	-	-

Dose of maintenance rapamycin (1 dose per week)	-	16 mg/kg per week	16 mg/kg per week		-	-	-
Ages, weekly rapamycin treatment	-	7–12 months	7–12 months		-	-	-

Dose of daily CCI-779 (5 days/week)	-	-	-		-	8 mg/kg per day	8 mg/kg per day
Ages, daily CCI-779 treatment	-	-	-		-	6–8 months	10–12 months

		This study				Messina et al., 2007	

* Tumor score from indicated group from Messina et al., 2007 compared with rapamycin group from this study

We selected the timing of rapamycin and IFN-g doses and schedules based on our prior findings showing treatment at 6–8 months or 10–12 months to be most effective using this model [20]. Rapamycin powder was obtained from LC Laboratories (Woburn, MA) and a 20 mg/ml stock of rapamycin was made in ethanol (stored at -20°C for up to one week). The stock solution was diluted to 1.2 mg/ml in vehicle (0.25% PEG, 0.25% Tween-80) for the 8 mg/kg dose and diluted to 2.4 mg/ml in vehicle for the 16 mg/kg dose. Murine IFN-g (R&D Systems, Minneapolis, MN) was diluted to 100,000 units/ml in sterile phosphate buffered saline (PBS) containing 0.1% mouse serum albumin (Sigma-Aldrich, Inc., St. Louis, MO) and stored at 4°C. All treatments were administered within 24 hours of making them. The health and behavior of all study animals were checked daily. Animals were weighed weekly, and at the time of necropsy, there were no significant differences in weight between cohorts. All experiments were done according to animal protocols approved by our institutional animal protocol review committee (Children's Hospital Boston, Boston, MA) and were compliant with federal, local, and institutional guidelines on the care of experimental animals.

### Quantification of kidney cystadenomas in *Tsc2*^+/- ^mice

For histological quantification of kidney cystadenomas, each kidney was fixed and sliced at 1 mm intervals. The kidney sections were then arranged sequentially for paraffin embedding, sectioning, and staining with hematoxylin and eosin (H&E). All slides were coded to keep scoring blinded, and all cystadenomas were counted, measured, and scored according to the scale shown in Table 2 by two blinded researchers (CW and AN). Cystadenomas that extended into more than one 1 mm kidney slice were counted only once and scored according to the maximum diameter.

**Table 2:** Scoring scale for kidney cystadenomas

Score	Area Range (mm^2^)
1	0.01 < x ≤ 0.09

2	0.09 < x ≤ 0.2

3	0.2 < x ≤ 0.35

4	0.35 < x ≤ 0.5

5	x > 0.5

Areas of cystadenomas were calculated by multiplying

the longest axis and its perpendicular axis.

Since the kidney cystadenomas of these *Tsc2*^+/- ^mice can be divided into subgroups including cystic, pre-papillary, papillary and solid lesions, we use "kidney cystadenomas" to refer to the entire spectrum of kidney lesions observed. In addition to analyzing data according to all cystadenomas, a subgroup analysis was also done by coding cystic, pre-papillary, papillary, and solid kidney lesions separately as indicated in Table 3. This is a slight modification to subgroup categories reported previously [20].

**Table 3:** Kidney Cystadenoma Subtypes

Lesion Subtype	Percentage Filled
Cystic	0%

Pre-papillary	0%< x < 25%

Papillary	25% ≤ x < 100%

Solid	100%

### Induction of subcutaneous *Tsc2*^-/- ^tumors in nude mice

Nude mice (strain CD-1nuBR, up to 6–8 weeks old) were obtained from Charles River Laboratories, Inc. (Wilmington, Massachusetts) and injected subcutaneously on the dorsal flank with 2.5 million NTC/T2null (*Tsc2*^-/-^*, Trp53*^-/-^) cells. As soon as tumors became visible, they were measured Monday through Friday using calipers. Tumor volumes were calculated using the formula: length × width × width × 0.5 [43]. All mice were euthanized once tumors reached ~3000 mm^3 ^in accordance with institutional animal care guidelines. Please note that survival analysis is done using time to tumor volume of ~3000 mm^3^, because this is when animals are euthanized. According to a protocol similar to our previous studies [18,20,21], data points for graphs of average tumor volume growth represent days when at least four mice in the indicated treatment group had tumor measurements. Statistical comparison of tumor volume measurements between groups is done on the last day that relevant groups had at least four tumor measurements.

### Treatment of subcutaneous tumors with sorafenib and rapamycin

Twenty-four CD-1 nude mice bearing *Tsc2*^-/- ^tumors were randomly assigned to one of four treatment arms: gavage vehicle (untreated control), rapamycin 8 mg/kg IP, sorafenib 60 mg/kg by gavage, or rapamycin 8 mg/kg IP plus sorafenib 60 mg/kg by gavage (see Table 4). Treatment was started once the tumors reached a volume of ~150 mm^3 ^(day 1 of treatment). Rapamycin treated mice received 200 μl of a 1.2 mg/ml solution of rapamycin daily (5 days per week, Monday through Friday) by IP injection. According to drug level testing, average rapamycin levels are ~12–40 ng/ml from 24–72 hours after a single 8 mg/kg dose of rapamycin. As trough levels for standard rapamycin dosing in humans is 3–20 ng/ml, the dosing used in these studies is relevant to rapamycin dosing in humans. Sorafenib treated mice received 60 mg/kg of sorafenib daily Monday through Friday by oral gavage. Sorafenib pills were obtained from the Brigham and Women's Hospital research pharmacy, crushed and diluted to make a 10 mg/ml suspension in 5% glucose for oral gavage stock. The sorafenib dose was based on preclinical studies in which daily oral administration of sorafenib at 30 to 60 mg/kg produced complete tumor stasis during treatment in five of six tumor models tested [34,44]. Rapamycin was prepared as previously described. The control group (gavage vehicle) received 200 μl of a 5% glucose solution daily Monday through Friday by oral gavage.

**Table 4:** Rapamycin plus sorafenib is more effective than single agent rapamycin in subcutaneous *Tsc2*^-/- ^tumors

	Untreated	Rapamycin	Sorafenib	Combination Rapamycin plus Sorafenib
Number of mice (n)	6	5	6	5

Median survival (days)	24.5	46	19.5	53
P value (survival)	-	0.0014*	NS*	0.0014* 0.0018^#^

Day 16, average tumor volume (mm^3^)	1454 ± 215	284 ± 52	2209 ± 499	293 ± 68
P value (day 16)	-	0.0007*	NS*	0.0009*

Day 43, average tumor volume (mm^3^)	-	2499 ± 222	-	1665 ± 245
P value (day 43)	-	-	-	0.036^#^

Day 44, average tumor volume (mm^3^)	-	2607 ± 247	-	1820 ± 245
P value (day 44)	-	-	-	0.06^#^

Rapamycin (5 days per week)	-	8 mg/kg per day	-	8 mg/kg per day
Sorafenib (5 days per week)	-	-	60 mg/kg per day	60 mg/kg per day

*compared to untreated

^#^compared to rapamycin treated

NS, not significant

The health and behavior of all mice were checked daily, and we did not observe significant toxicity from treatment with rapamycin, sorafenib, or the combination of rapamycin plus sorafenib at the doses used in this study. Once tumors reached the endpoint volume of ~3000 mm^3^, the mice were sacrificed. Upon sacrifice, whole blood and tumor tissue were harvested. Mice were weighed on day one of their treatment and at necropsy; no notable changes were seen in any cohorts (data not shown).

Two mice were excluded from the analyses. One mouse assigned to the rapamycin 8 mg/kg daily IP group was euthanized due to weight loss and dehydration prior to starting any drug treatments. Another mouse assigned to rapamycin 8 mg/kg plus sorafenib 60 mg/kg daily treatment was removed from study due to an extremely slow growing tumor that did not reach treatment threshold volumes. Both mice that were excluded did not start any treatments prior to euthanasia so their conditions were unrelated to study treatments. All drug doses were calculated based on an average weight of 30 g per mouse.

### Treatment of subcutaneous tumors with atorvastatin, doxycycline, and rapamycin

To determine if atorvastatin or doxycycline are useful therapeutic drugs for TSC, the efficacy of atorvastatin and doxycycline as single agents and in combination with rapamycin were tested in the subcutaneous tumor model for TSC-related tumors. A cohort of 48 CD-1 nude mice was injected with NTC/T2null (*Tsc2*^-/-^*, Trp53*^-/-^) cells. The cohort was then divided into 6 randomly assigned groups: untreated control group, single agent rapamycin, atorvastatin, combination atorvastatin plus rapamycin, single agent doxycycline, and combination doxycycline plus rapamycin (see Table 5).

**Table 5:** Summary of atorvastatin and doxycycline preclinical data

	Untreated	Rapamycin	Atorvastatin	Combination Rapamycin plus Atorvastatin	Doxycycline	Combination Rapamycin plus Doxycycline
Number of mice (n)	8	8	8	8	8	8

Median survival (days)	28	41.5	32.5	55.5	28	43
P value (survival)	-	0.003*	NS*	0.0006* NS^#^	NS*	0.007* NS^#^

Day 26, average tumor volume (mm^3^)	1865 ± 532	544 ± 110	1931 ± 298	390 ± 186	1749 ± 352	1117 ± 421
P value (day 26)	-	0.011*	NS*	0.04*	NS*	NS*

Day 42, average tumor volume (mm^3^)	-	1784 ± 643	-	1188 ± 338	-	2419 ± 570
P value (day 42)	-	-	-	NS^#^	-	NS^#^

Rapamycin (IP, 3 days per week)	-	8 mg/kg, 3 days per week	-	8 mg/kg, 3 days per week	-	8 mg/kg, 3 days per week
Atorvastatin (IP, 5 days per week)	-	-	20 mg/kg per day	20 mg/kg per day	-	-
Doxycycline (IP, 5 days per week)	-	-	-	-	10 mg/kg per day	10 mg/kg per day

*compared to untreated

^#^compared to rapamycin treated

NS, not significant

All drug treatments started when tumors reached a volume of ~50 mm^3 ^(day 1 of treatment), regardless of treatment schedule, and animals were euthanized when tumors reached a volume of ~3000 mm^3^. If a volume of 40 mm^3 ^was reached on Thursday or Friday, treatment began that day. Otherwise, treatment was started on the day tumor volume was ≥50 mm^3^. Untreated mice did not receive any treatment even after tumors reach a volume ≥50 mm^3^. Please note that this is a minor difference in study design from the sorafenib study. We have previously shown that differences in tumor volume at the start of treatment are not likely to have any major impact on efficacy [20].

Rapamycin treated groups received 200 μl of a 1.2 mg/ml solution of rapamycin (8 mg/kg) three times per week (on Mondays, Wednesdays and Fridays) by IP injection. Mice being treated with doxycycline were treated daily Monday through Friday with 200 μl of a 1.5 mg/ml (10 mg/kg) IP injection. Atorvastatin groups received 200 μl daily of a 3 mg/ml solution (20 mg/kg) by IP injection Monday through Friday. All drug doses were calculated based on an average weight of 30 g per mouse. Atorvastatin powder was obtained from LKT Laboratories, Inc. (St. Paul, MN) and was diluted in 1% ethanol in sterile PBS. This dose of atorvastatin was based on a study [45] in which this dose was effective in reducing atherosclerotic lesions in a mouse model. Doxycycline powder was obtained from Sigma-Aldrich Co. (St. Louis, MO) and was diluted in sterile PBS. This 10 mg/kg dose of doxycycline was based on a study of the efficacy of minocycline and doxycycline in treating Huntington's Disease, which showed the dose to be biologically active but not effective in treating Huntington's Disease [46]. Rapamycin preparation was described above.

Once tumors reached the endpoint volume of ~3000 mm^3^, the mice were sacrificed. Animal behavior and health were monitored daily, and animals were weighed at the start of the study and at the time of necropsy. While there were no significant differences in weight at necropsy between cohorts, all mice receiving rapamycin failed to gain weight as other cohorts do (Table 6). We did not observe other evidence of toxicity from treatment with rapamycin, atorvastatin, doxycycline, or combinations at the doses used in this study. All mice from rapamycin treated cohorts were euthanized 24 hours after the last rapamycin treatment upon reaching the endpoint tumor volume. Upon sacrifice, whole blood and tumor were harvested for drug level testing.

**Table 6:** Rapamycin treated nude mice with *Tsc2*^-/- ^tumors fail to gain weight

	Weight at Start (g)	Weight at Necropsy (g)	Weight gain (g)	P value
Untreated	34.6 ± 1.1	37.8 ± 0.9	3.2 ± 0.9	*0.0388
				-
				-

Rapamycin treated	36.0 ± 0.6	35.6 ± 0.6	-0.4 ± 0.6	*NS
				**NS
				^#^NS

Other treatment	33.8 ± 0.6	36.8 ± 0.7	3.1 ± 0.4	*0.0024
				**NS
				^#^NS

*Weight at start compared with weight at necropsy (within the same treatment group)

**Weight at start compared to untreated at start

^#^Weight at necropsy compared to untreated at necropsy

### Whole blood and tumor rapamycin levels

Whole blood or tumor rapamycin levels were measured from a subset of animals treated with rapamycin in the nude mouse treatment studies described above. Blood and tumors were harvested at necropsy 24 hours after the final treatment of rapamycin. Tumor samples were prepared by homogenizing 200 mg of tumor tissue in 1 ml of sterile PBS. Whole blood was obtained through cardiac puncture, dispensed into an EDTA-containing blood collection tube, and diluted with an equal volume of sterile phosphate buffered saline to ensure sufficient volume for rapamycin level analysis. All measured rapamycin levels were then corrected according to sample dilution at time of analysis. Tumor samples and whole blood samples were tested for rapamycin levels at the Clinical Laboratory at Children's Hospital Boston (Boston, Massachusetts). The range of detection is 0.5 to 100 ng/ml of rapamycin.

### Statistical analyses

GraphPad Prism software (version 4.01) was used for all data analysis, with p-value ≥ 0.05 indicating statistical significance. All calculations were completed from raw data by three authors (NL, AN, and CW) and verified with calculations from two other authors (AR and MM). A standard unpaired *t *test was used to test all quantitative data, and the Mantel-Cox logrank analysis was used for survival data, which is defined as time to reach a tumor volume of ~3000 mm^3^.

## Results

### Comparison of rapamycin with combination rapamycin plus IFN-g in *Tsc2*^+/- ^mice treated using a schedule that includes daily dosing and weekly maintenance therapy

In prior studies, combination therapy was more effective than single agent CCI-779 in the treatment of nude mice bearing *Tsc2*^-/- ^tumors, but we saw no difference between these groups in the *Tsc2*^+/- ^kidney tumor model. In order to further evaluate the potential benefits of mTOR inhibitor plus IFN-g combination therapy in the *Tsc2*^+/- ^kidney tumor model, we compared single agent rapamycin treatment to rapamycin plus IFN-g treatment using a dosing schedule that includes daily treatment (five days per week) for one month before and after a period of weekly maintenance treatment for five months.

**Figure 1:**
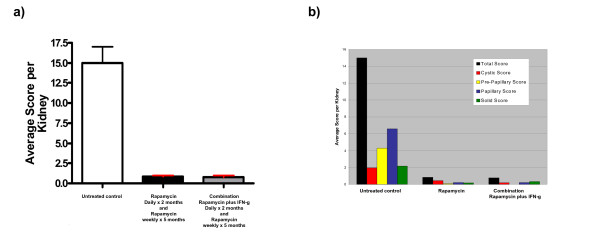
**Comparison of single agent rapamycin treatment to rapamycin plus IFN-g treatment in *Tsc2*^+/- ^mice using a dosing schedule that includes weekly maintenance rapamycin treatment**. Severity of kidney disease in *Tsc2*^+/- ^mice was quantified by scoring (a) total kidney cystadenomas and (b) kidney lesion subtype for indicated treatment groups (see treatment details in methods and Table 1). Lesion subtypes include cystic, pre-papillary, papillary, and solid; for a definition of each subtype and scoring details, see Tables 2 and 3. The red error bars in (a) indicate a statistically significant difference (p < 0.05) relative to the untreated cohort. Statistical analysis was not done for the lesion subtype date. These data show a significant reduction of kidney disease in both treatment groups but no difference between single agent rapamycin and combination rapamycin plus IFN-g. Error bars shown indicate standard error.

Relative to the untreated cohort, both treatment groups showed a significantly lower disease burden as evaluated by kidney cystadenoma score (Figure 1a). No significant difference was observed in kidney cystadenoma score between the rapamycin treated cohort and the combination treated cohort. This result is similar to the finding we reported in Messina et al. 2007 in a *Tsc2*^+/- ^mouse study, but differs from our observation using the subcutaneous *Tsc2*^-/- ^tumor model [19]. In this case, we note that the single agent rapamycin treatment group was extremely effective and reduced kidney disease by 94.5% compared with untreated controls.

We also analyzed this data according to kidney lesion subtype (Figure 1b). All *Tsc2*^+/- ^kidney lesions can be subdivided into four categories: cystic lesions, pre-papillary lesions, papillary lesions, and solid lesions. Cystadenomas were scored according to lesion subtype (see Table 3) to investigate the impact of treatment on lesion subtype as well as document the distribution of these subtypes in untreated animals. Papillary lesions were the most common subtype in untreated *Tsc2*^+/- ^mice while cystic and solid lesions were the least common. Cystic lesions were most common in the rapamycin treated cohort, and solid lesions appeared most often in the rapamycin and IFN-g combination treated cohort. Treatment with rapamycin alone or combination rapamycin plus IFN-g reduced the score of all subtypes of kidney lesions.

### Combination of rapamycin plus sorafenib (a VEGF pathway inhibitor) is more effective than single agent rapamycin

In order to evaluate whether inhibition of VEGF signaling is a useful therapeutic strategy for the treatment of TSC-related tumors, we investigated the efficacy of sorafenib as a single agent and in combination with rapamycin in treating a relevant subcutaneous tumor model (see methods). We used nude mice bearing subcutaneous *Tsc2*^-/- ^tumors derived from NTC/T2null (*Tsc2*^-/-^*, Trp53*^-/-^) cells with the following cohorts: untreated controls, rapamycin treated, sorafenib treated, and sorafenib plus rapamycin combination treated.

**Figure 2:**
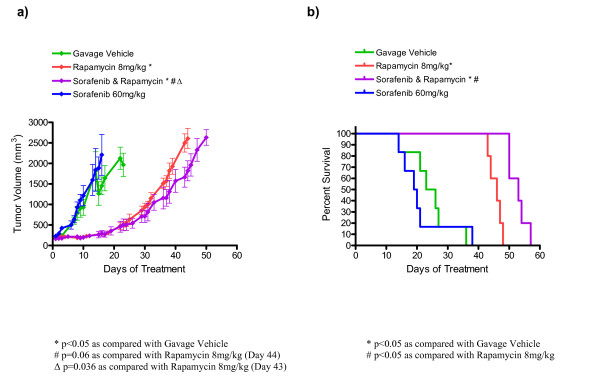
**Improved survival and decreased tumor growth in nude mice bearing *Tsc2*^-/- ^tumors with combination rapamycin plus sorafenib treatment**. (a) Average tumor volume over time. Error bars shown indicate standard error. (b) Survival curve for indicated treatment cohorts. Based on survival analysis, rapamycin plus sorafenib is more effective than single agent rapamycin. Based on tumor volume analyses, the rapamycin plus sorafenib treated group is not statistically different from the rapamycin treated group on day 44. However, on the last day that tumor volume data was available for all mice (day 43), there was a significant difference between tumor volumes from the rapamycin plus sorafenib group compared with the single agent rapamycin group. Although rapamycin is an effective single agent, sorafenib is not. This data is summarized in Table 4.

Average tumor growth is shown for each treatment group in Figure 2a and Table 4. According to our protocol, the data points shown represent days when at least four mice of the treatment group were treated and had tumors measured (see Methods). We compared tumor volumes of single agent treatment to untreated controls on day 16 because that was the last day that all three groups (untreated, rapamycin, sorafenib) had at least four tumor measurements. Consistent with our prior studies, the rapamycin treated group had a significantly lower tumor volume than the untreated group (284 ± 52 mm^3 ^for rapamycin, 1454 ± 215 mm^3 ^for untreated, p = 0.0007). Single agent sorafenib was not effective as the day 16 tumor volume was 2209 ± 499 mm^3^, which is not significantly different from the untreated control group. Survival analysis comparing single agent treatment to untreated controls was in agreement with the tumor volume comparisons.

We also compared combination sorafenib plus rapamycin with single agent rapamycin treatment to evaluate the potential utility of VEGF pathway plus mTOR pathway inhibition. Comparing survival curves (see Figure 2b and Table 4) using the Mantel Cox logrank analysis, we observed improved survival in the combination sorafenib plus rapamycin treatment group (median survival of 53 days) compared with the rapamycin treatment group (median survival of 46 days, p = 0.0018). We also compared tumor volumes in these two groups. According to our protocol, we compared tumor volumes on treatment day 44 (that was the last day that both groups had at least four tumor measurements) and found the average tumor volume of the rapamycin plus sorafenib treated group (1820 ± 245 mm^3^, n = 5) was smaller than the average tumor volume of the rapamycin treated group (2607 ± 247 mm^3^, n = 4); this difference approaches statistical significance (p = 0.06). In this case, we also compared tumor volumes on day 43 when there were tumor measurements for all mice in both groups, the difference was statistically significant (1665 ± 245 mm^3 ^for rapamycin plus sorafenib; 2499 ± 222 mm^3 ^for single agent rapamycin, p = 0.036).

### Atorvastatin as a single agent or in combination with rapamycin does not decrease tumor burden or increase survival in nude mice bearing *Tsc2*^-/- ^tumors

**Figure 3:**
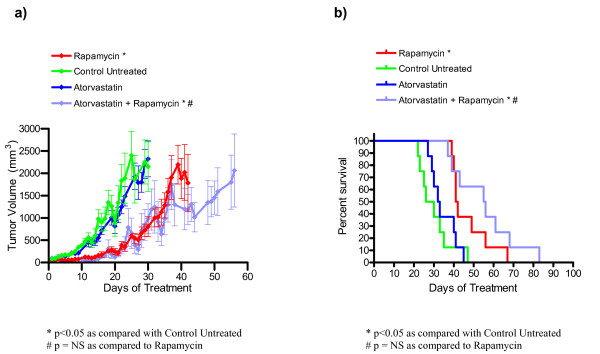
**Atorvastatin does not decrease tumor growth or increase survival in nude mice bearing *Tsc2*^-/- ^tumors**. (a) Average tumor growth over time for atorvastatin and atorvastatin plus rapamycin treated animals. Error bars shown indicate standard error. (b) Survival over time for atorvastatin and atorvastatin plus rapamycin cohorts. Based on survival analysis and comparison of tumor volumes on days 26 and 42, atorvastatin was not effective as a single agent or in combination with rapamycin in this preclinical study. This data is summarized in Table 5.

As shown in Figure 3 and Table Table 5, atorvastatin did not reduce tumor growth or improve survival as a single agent. Furthermore, adding atorvastatin to rapamycin did not reduce disease severity when compared with single agent rapamycin treatment. Data points for average tumor volume (Figure 3a) are included on days where at least four of the animals in a cohort had tumors measured. The day 26 average tumor volume (mean ± standard error) was 544 ± 110 mm^3 ^for the rapamycin group and 390 ± 186 mm^3 ^for atorvastatin plus rapamycin. These were significantly lower than the day 26 average tumor volume for the untreated cohort (1865 ± 532 mm^3^). In contrast, the day 26 average tumor volume for single agent atorvastatin (1931 ± 298 mm^3^) was not significantly different than the untreated group. The day 26 average tumor volume for single agent atorvastatin was significantly higher than the rapamycin cohort, while the average tumor volume for atorvastatin plus rapamycin did not differ significantly from the average tumor volume for the single agent rapamycin cohort.

At day 42, the average tumor volume for atorvastatin plus rapamycin group (1188 ± 338 mm^3^) was not significantly lower than the single agent rapamycin cohort (1784 ± 643 mm^3^). Survival data from this experiment is shown in Figure 3b and Table 5. We observed a significant improvement in median survival for both the rapamycin group (41.5 days, p = 0.003) and the atorvastatin plus rapamycin group (55.5 days, p = 0.0006) when compared to the untreated cohort (28 days). However, the median survival between the rapamycin treated group and the atorvastatin plus rapamycin treated group was not significantly different. Although the median survival of atorvastatin treated animals (32.5 days) was slightly longer than in the untreated cohort, this difference was also not statistically significant. In summary, the survival data together with the tumor volume data demonstrate that we did not observe any benefit to adding atorvastatin to rapamycin treatment in this preclinical TSC tumor model.

### Doxycycline as a single agent or in combination with rapamycin does not decrease tumor burden or increase survival in nude mice bearing *Tsc2*^-/- ^tumors

**Figure 4:**
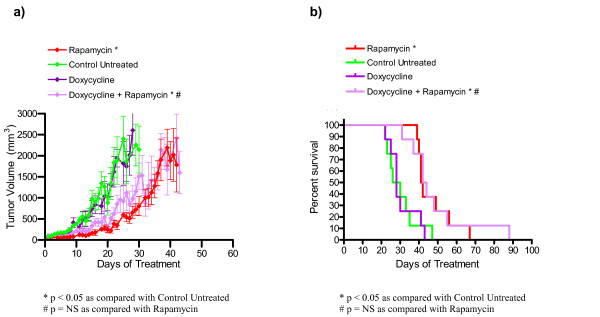
**Doxycycline does not decrease tumor growth or increase survival in nude mice bearing *Tsc2*^-/- ^tumors**. (a) Average tumor growth over time for doxycycline and doxycycline plus rapamycin treated animals. Error bars shown indicate standard error. (b) Survival over time for doxycycline and doxycycline plus rapamycin cohorts. Based on survival analysis and comparison of tumor volumes on days 26 and 42, doxycycline was not effective as a single agent or in combination with rapamycin in this preclinical study. This data is summarized in Table 5.

Tumor volume and survival data for the doxycycline treated mice along with rapamycin treated and untreated control group are shown in Figure 4 and Table 5. Figure 4a shows average tumor growth over time for the doxycycline treated animals. The data points represent days where at least four of the animals in a cohort had tumors measured. The day 26 average tumor volumes (mean ± standard error) for the single agent doxycycline cohort (1749 ± 352 mm^3^) and the doxycycline plus rapamycin treated animals (1117 ± 421 mm^3^) were not significantly different than the untreated group (1865 ± 532 mm^3^). The average tumor volume for doxycycline plus rapamycin (2419 ± 570 mm^3^) was similar to the rapamycin cohort (1784 ± 643 mm^3^) at day 42 (Figure 4a, Table 5), and survival data for the doxycycline experiment was consistent with the tumor volume data (Figure 4b). The median survival of the doxycycline plus rapamycin treated cohort (43 days, p = 0.007) was significantly increased compared to the untreated cohort (28 days) but was similar to rapamycin treated animals. The median survival of the doxycycline cohort (28 days) was not significantly different than the untreated cohort. In summary, doxycycline was not effective as either a single agent or in combination with rapamycin in this preclinical model for TSC-related tumors.

### Sorafenib, atorvastatin and doxycycline do not affect rapamycin levels in combination treatment cohorts

**Figure 5:**
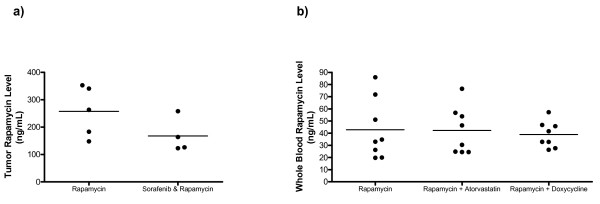
**Sorafenib, atorvastatin and doxycycline do not affect whole blood rapamycin levels in nude mice bearing *Tsc2*^-/- ^tumors**. (a) Tumor rapamycin levels from rapamycin treated group (n = 5) and sorafenib plus rapamycin combination treated group (n = 4). (b) Whole blood rapamycin levels from rapamycin treated group (n = 8), atorvastatin plus rapamycin combination treated group (n = 8) and doxycycline plus rapamycin combination treated group (n = 8). Rapamycin levels were measured 24 hours after the last dose of rapamycin for all groups.

Rapamycin is metabolized by CYP3A4 so rapamycin levels can vary when there is exposure to other drugs that either induce or inhibit CYP3A4 [47]. To be sure there were no significant drug interaction issues in our studies, rapamycin levels were measured in tumors or whole blood 24 hours after the last dose in a subset of animals from our studies (Figure 5). Average tumor rapamycin levels (mean ± standard deviation) in the sorafenib plus rapamycin treated group (167.8 ± 63.1 ng/ml) and the rapamycin treated group (257.6 ± 91.6 ng/ml) were not statistically different (p = 0.14, NS). Average blood rapamycin levels (mean ± standard deviation) from neither the atorvastatin plus rapamycin group (42.2 ± 19.3 ng/ml, p = 0.96, NS) nor the doxycycline plus rapamycin group (38.9 ± 10.8 ng/ml, p = 0.68, NS) were statistically different from the average blood rapamycin level of the single agent rapamycin group (42.9 ± 24.7 ng/ml). We have previously observed higher 24 hour rapamycin levels in tumor tissue when compared with blood [21] so the differences in tumor versus blood levels shown in Figure 5 are consistent with our prior results. Based on drug level testing, we conclude that sorafenib, atorvastatin, and doxycycline did not significantly affect the metabolism of rapamycin in the preclinical studies reported here.

## Discussion

In prior preclinical studies, we used two *TSC2 *tumor models to demonstrate that while both the rapamycin analog, CCI-779, and IFN-g are effective in reducing tumor growth, rapamycin is more effective than CCI-779, and effective rapamycin doses are absorbed after topical administration [18-21]. We also investigated the optimal timing of treatment [20] using these models. We observed conflicting results regarding whether treatment with an mTOR inhibitor plus IFN-g is better than an mTOR inhibitor as a single agent [19,20].

The preclinical studies reported here were done to address questions relevant to the design of future TSC clinical trials. One goal of the *Tsc2*^+/- ^experiment was to compare the combination of rapamycin plus IFN-g to single agent rapamycin using a dosing schedule for rapamycin that included daily treatment and weekly treatment. Although we did not see any benefit to the addition of IFN-g, we also noted that the rapamycin single agent treatment was very effective. We observed a dramatic 94.5% reduction in tumor burden in *Tsc2*^+/- ^mice treated with one month of daily rapamycin treatment before and after five months of weekly rapamycin therapy. Although IFN-g clearly has activity in some of our prior studies [18,19], we observed that IFN-g seems to be effective when given for a prolonged period of time and is not as effective when given only short term (this study and Messina et al. 2007). In this study, the single agent rapamycin treatment was so effective that it would be difficult to improve on the 94.5% reduction in kidney disease severity that was observed.

This dramatic result in the rapamycin single agent group prompted us to review our prior studies. As illustrated in Table 7, we see a 94.5% reduction in kidney disease in *Tsc2*^+/- ^mice treated with daily rapamycin for one month before and after weekly rapamycin for five months in this study. In contrast, two months of daily CCI-779 without maintenance therapy was effective but only reduced disease severity by 64.5% [20]. A comparison of the *Tsc2*^+/- ^preclinical results from Messina et al., 2007 to this study is summarized in Table 1. Treatment with rapamycin (daily for one month followed by weekly for five months and then daily for the last month, this study) showed significantly lower tumor burden than both the 6–8 months and 10–12 months CCI-779 treated cohorts from Messina et al. (p < 0.0001; p < 0.0001) [20].

**Table 7:** Comparison of *Tsc2*^+/- ^mTOR inhibitor trials

**mTOR inhibitor**	**Total drug dose (per mouse)**	**Schedule**	**% Reduction in kidney tumors**	**Study**
Rapamycin	19.2 mg	Part one: 8 mg/kg daily × 1 month	94.5%*	This study
		Part two: 16 mg/kg weekly × 5 months		
		Part three: 8 mg/kg daily × 1 month		

CCI-779	9.6 mg	8 mg/kg daily × 2 months	63%*	Messina et al. 2007
			64%**	

CCI-779	4.32 mg	4 mg/kg 3 times a week × 3 months	83.6%**	Lee et al. 2005

* Kidney tumor score

**Kidney tumor number

In Messina et al. (2007), we showed that the severity of kidney disease increases with an increase in age in untreated *Tsc2*^+/- ^mice [20]. It is interesting to point out that the CCI-779 treated cohorts were evaluated for severity of kidney disease at 12 months of age (Messina et al. 2007), and rapamycin treated cohorts were evaluated at 13 months of age (this study). According to our previous data on the genesis of kidney disease at different ages, the mice euthanized at 13 months of age should have a higher severity of kidney disease than those euthanized at 12 months of age. Untreated *Tsc2*^+/- ^mice euthanized at 12 months were found to have an average score per kidney of 9.95 ± 1.59 while untreated *Tsc2*^+/- ^mice euthanized at 13 months were found to have an average score per kidney of 15.00 ± 2.01. The observation that the older rapamycin treated cohort (0.83 ± 0.18 average score per kidney) showing less tumor burden than the younger CCI-779 treated cohorts (treated 6–8 months, average score per kidney 6.00 ± 1.01; treated 10–12 months, average score per kidney 3.54 ± 0.76) is even more striking when this study design difference is considered.

While prior studies also examined mTOR inhibitor efficacy in treating TSC-related kidney lesions, several inter-study differences are limitations that prevent rigorous comparisons. One difference between this study and Messina et al. (2007) is that different mTOR inhibitors were used. Although rapamycin and CCI-779 are similar, we have recently shown that rapamycin (8 mg/kg IP 5 days per week) is more effective than CCI-779 (8 mg/kg IP 5 days per week) in the *Tsc2*^-/- ^subcutaneous tumor model, raising the possibility that the difference is due to rapamycin's higher efficacy as compared to CCI-779 rather than the addition of prolonged weekly maintenance dosing. Interestingly, when we compare data from two prior CCI-779 studies [18,20], we noted that CCI-779 given at a lower dose 3 times per week for 3 months is more effective than CCI-779 given daily for 2 months (84% reduction in Lee et al. 2005 versus 64% reduction in Messina et al. 2007, see Table 7). This is somewhat surprising as the total CCI-779 dose per mouse used in Lee et al. 2005 (4.32 mg) is lower than in Messina et al. 2007 (9.6 mg). Possible minor strain variation between the *Tsc2*^+/- ^mice used in the different studies is another potential difference that limits rigorous direct comparisons. Despite the study differences, taken together, our observations suggest that lower doses of an mTOR inhibitor for a longer duration may be more effective in TSC preclinical models. This will be further investigated and may have implications for future TSC clinical trials.

In early clinical studies, rapamycin treatment causes TSC-related tumor regression [23,24]. Because this tumor regression is incomplete and responses are not durable [24], there is significant interest in identifying novel agents for TSC-related tumors to be used either as single agents or in combination with rapamycin. In this study, we evaluated three novel drug classes: a multi-targeted kinase inhibitor (sorafenib), a statin (atorvastatin), and an MMP inhibitor (doxycycline) as single agents and in combination with rapamycin. We found that combination sorafenib plus rapamycin was more effective than rapamycin according to survival analysis, but the difference was not dramatic and we were surprised by the lack of benefit of single agent sorafenib. Limitations of this study include the small numbers in each treatment group and that only a single dose of sorafenib was tested. It is possible that single agent sorafenib may be effective at higher doses or earlier treatment. Because of the potential for an effect due to drug interactions, we measured rapamycin levels and found that there was no significant difference in rapamycin levels in the presence or absence of sorafenib treatment. In our sorafenib plus rapamycin experiment, although the improvements were not dramatic, it was statistically significant for survival analysis and approached statistical significance for tumor volume analysis on day 44. While the improvements in tumor size were not statistically significant on day 44, it is important to note that these improvements were statistically significant when comparing the groups on day 43 when both cohorts had all five assigned mice. By day 44, a rapamycin treated mouse had reached a tumor volume of ~3000 mm^3 ^and had been sacrificed so that it was not included in the day 44 tumor volume analysis. Because our protocol incorporates this bias against finding a difference between rapamycin treated and combination treated groups by excluding measurements of tumor volumes beyond ~3000 mm^3 ^(when mice must be euthanized), the data presented here suggest that further study into VEGF inhibitors in combination with rapamycin is warranted. Furthermore, this data is consistent with other published data showing that VEGF signaling is important in TSC disease pathogenesis. Based on these positive findings, we are enthusiastic about further investigating VEGF signaling in TSC & LAM pathogenesis and additional TSC preclinical studies evaluating other VEGF pathway inhibitors as well as different schedules and dosing of the combination of VEGF inhibitors plus rapamycin.

In contrast, doxycycline (an MMP inhibitor) and atorvastatin (a HMG-CoA reductase inhibitor) were not effective as single agents or in combination with rapamycin. A limitation of this study is that only one dose was tested, so it is possible that a higher dose or different schedule could alter these results. Another limitation is that tumor cells for subcutaneous injection into nude mice were *p53 null *in addition to *Tsc2*^-/-^. We submitted blood samples for rapamycin level testing to be sure that there was no evidence of significant drug interaction issues. Although our findings are not consistent with in vitro studies showing that atorvastatin inhibits the proliferation of *Tsc2*^-/- ^cells [37] and doxycycline decreases invasiveness of cells derived from LAM tissue [39], these studies were done using cultured cells, which is an important difference. Based on our findings, we are not enthusiastic about pursuing further preclinical studies or clinical trials with these drug classes.

## Conclusion

The results of the preclinical studies reported here indicate that prolonged exposure to relatively low doses of mTOR inhibitors may be a useful strategy to achieve more durable responses of TSC-related tumors and should be pursued in further preclinical studies and TSC trials. Furthermore, survival data in a TSC preclinical model suggests that the combination of rapamycin plus sorafenib, a multi-targeted kinase inhibitor that targets the VEGF pathway, may be more effective than single agent rapamycin. This finding has implications for evaluation of other angiogenesis and multi-targeted kinase inhibitors in future TSC preclinical studies and demonstrates that targeting multiple signaling pathways may be a useful strategy for the treatment of TSC.

## Abbreviations

TSC: Tuberous Sclerosis Complex; mTOR: Mammalian target of rapamycin; IFN-g: Interferon-gamma; VEGF: Vascular endothelial growth factor; LAM: lymphangioleiomyomatosis; Rheb: Ras homologue enriched in brain; mTORC1: Mammalian target of rapamycin complex 1; SEGA: Subependymal giant cell astrocytoma; SGCT: Subependymal giant cell tumor; HIF: Hypoxia induced factor; VEGFR: Vascular endothelial growth factor receptor; PDGFR: Platelet derived growth factor receptor; FLT-3: fms-related tyrosine kinase 3; MMP: Matrix metalloproteinase; LOH: Loss of heterozygosity; IP: intraperitoneal; PEG: Polyethylene glycol; PBS: Phosphate buffered saline; EDTA: ethylenediaminetetraacetic acid; kg: kilogram; g: gram; ng: nanogram; mg: milligram; ml: milliliter; μl: microliter; mm: millimeter

## Authors' contributions

NL assisted with experimental design and data collection, performed statistical analyses, wrote the majority of the first draft of the manuscript, and helped edit the manuscript. CW assisted with experimental design and data collection, performed statistical analyses, and wrote parts of the first draft of the manuscript. AN assisted with experimental design and data collection, performed statistical analyses, and wrote parts of the first draft of the manuscript. AR assisted with experimental design and data collection, and performed independent verification of statistical analyses. MM assisted with experimental design and data collection, and performed some statistical analyses. SD provided funding, critical guidance for the experiments, and was responsible for supervising the writing and editing of the manuscript. All authors have read and approved this manuscript.
